# *CaMK4* Gene Deletion Induces Hypertension

**DOI:** 10.1161/JAHA.112.001081

**Published:** 2012-08-24

**Authors:** Gaetano Santulli, Ersilia Cipolletta, Daniela Sorriento, Carmine Del Giudice, Antonio Anastasio, Sara Monaco, Angela Serena Maione, Gianluigi Condorelli, Annibale Puca, Bruno Trimarco, Maddalena Illario, Guido Iaccarino

**Affiliations:** Department of Clinical Medicine, Cardiovascular and Immunologic Sciences, “Federico II” University of Naples, Naples, Italy (G.S., E.C., D.S., C.D.G., A.A., B.T.); Department of Cellular and Molecular Biology and Pathology, “Federico II” University of Naples, Naples, Italy (S.M., A.S.M., M.I.); Istituto clinico Humanitas IRCCS and Istituto Ricerca Genetica Biomedica, National Research Council, Rozzano, Italy (G.C.); Multimedica Research Hospital, Milan, Italy (G.C., A.P., G.I.); Department of Medicine and Surgery, University of Salerno, Salerno, Italy (A.P., G.I.)

**Keywords:** angiogenesis, arrhythmia, endothelium, hypertension, hypertrophy

## Abstract

**Background:**

The expression of calcium/calmodulin-dependent kinase IV (CaMKIV) was hitherto thought to be confined to the nervous system. However, a recent genome-wide analysis indicated an association between hypertension and a single-nucleotide polymorphism (rs10491334) of the human CaMKIV gene (*CaMK4)*, which suggests a role for this kinase in the regulation of vascular tone.

**Methods and Results:**

To directly assess the role of CaMKIV in hypertension, we characterized the cardiovascular phenotype of *CaMK4*^−/−^ mice. They displayed a typical hypertensive phenotype, including high blood pressure levels, cardiac hypertrophy, vascular and kidney damage, and reduced tolerance to chronic ischemia and myocardial infarction compared with wild-type littermates. Interestingly, in vitro experiments showed the ability of this kinase to activate endothelial nitric oxide synthase. Eventually, in a population study, we found that the rs10491334 variant associates with a reduction in the expression levels of CaMKIV in lymphocytes from hypertensive patients.

**Conclusions:**

Taken together, our results provide evidence that CaMKIV plays a pivotal role in blood pressure regulation through the control of endothelial nitric oxide synthase activity. **(*J Am Heart Assoc*. 2012;1:e001081 doi: 10.1161/JAHA.112.001081.)**

## Introduction

A growing body of evidence bears out the rising interest in the function of calcium/calmodulin-dependent kinases (CaMKs) in cardiovascular pathophysiology. In particular, although it is now established that CaMKII is an important player in the regulation of cardiac responses, both in terms of electrophysiology and of cardiac myocyte hypertrophy,^1–4^ less is known about the role of other members of the CaMK family, such as CaMKIV, in the cardiovascular system.^5^

The recent genome-wide analysis of the Framingham Heart Study 100K Project^6^ showed an association between elevated diastolic blood pressure (BP) and the rs10491334 T/C single-nucleotide polymorphism (SNP) of the human CaMKIV gene (*CaMK4*). Such a finding suggests that this kinase, the expression of which once was thought to be confined to the nervous tissue,^7–10^ has a yet unidentified role in the control of vascular tone.

We therefore hypothesized that CaMKIV could affect endothelial functions, such as the control of vascular resistance, and that changes in its level of expression or activity in endothelial cells (ECs) might alter the fine regulation of vascular responses, causing hypertension. To ascertain whether CaMKIV signaling is involved in endothelial dysfunction, a hallmark of the hypertensive state,^11–14^ we used a murine model of genetic deletion of *CaMK4*. Finally, a population study was carried out in normotensive and hypertensive patients to investigate the effects of the *CaMK4* rs10491334 SNP in humans.

## Methods

### In Vivo Studies

#### Animals

All animal procedures were performed in accordance with the policies and guidelines of the “Position of the American Heart Association on Research Animal Use”^15^ and were approved by the Ethics Committee of the “Federico II” University. The European Commission Directive 2010/63/EU was followed. We studied male mice with global homozygous deletion of the *CaMK4* gene (*CaMK4*^−/−^), backcrossed >12 generations onto a C57Bl/6J background. The mice were kindly provided by Anthony Means (Duke University, Durham, NC).^9^ Age-matched wild-type littermates (*CaMK4*^+/+^) were used as controls. The animals were housed in a 22°C room with a 12-hour light/dark cycle and were allowed food and tap water ad libitum. Two groups of mice (*CAMK4*^+/+^ and *CAMK4*^−/−^) were subjected to a long-term furosemide treatment (0.35 mg/kg per day) from the age of 3 months until 6 or 18 months. The drug was added directly to the drinking water. The development of the typical target-organ damage (vascular, cardiac, and kidney damage)^16–18^ was evaluated in 18-month-old mice. Longitudinal survival observation on *CAMK4*^+/+^ and *CAMK4*^−/−^ mice was performed over a period of 24 months. Genotypes were determined by polymerase chain reaction amplification of tail DNA. The individual performing all experiments was blinded to the mouse genotype until all data were fully analyzed.

#### Invasive Arterial BP Measurement

Mice were anesthetized by isoflurane (4%) inhalation and maintained by mask ventilation (isoflurane 1.8%). Direct BP and heart rate measurements were performed with the use of a 1.0F Mikro-Tip catheter (SPR1000, Millar Instruments, Houston, TX), which was advanced through the right external carotid artery and placed in the descending aorta. After implantation, the catheter was connected to a transducer (Gould Instruments Systems, Cleveland, OH) to record BP and heart rate for 15 minutes. The pressure catheter then was advanced through the aortic valve into the left ventricle (LV). Subsequent offline evaluation provided the first derivative of the LV pressure curve (maximum and minimum d*P*/d*t*). All data were analyzed with dedicated software (PowerLab-Chart 7.1, ADInstruments, Sydney, Australia).

#### Echocardiography

Transthoracic echocardiography was performed with a small-animal high-resolution imaging system (VeVo770, VisualSonics, Inc, Toronto, Canada) equipped with a 30-MHz transducer (Real-Time Micro Visualization, RMV-707B). The mice, anesthetized as described previously for BP measurement, were placed in a shallow left lateral decubitus position, with strict thermoregulation (37±1°C) to optimize physiological conditions and reduce hemodynamic variability. Fur was removed from the chest by application of a cosmetic cream (Veet, Reckitt Benckiser, Milan, Italy) to gain a clear image. LV end-diastolic and LV end-systolic diameters were measured at the level of the papillary muscles from the parasternal short-axis view.^19–20^ Intraventricular septal and LV posterior wall thickness were estimated at end diastole. LV fractional shortening was calculated as follows: LVFS = [(LVEDD − LVESD)/ LVEDD] × 100, where LVFS indicates LV fractional shortening; LVEDD, LV end-diastolic diameter; and LVESD, LV end-systolic diameter. LV ejection fraction was calculated automatically by the echocardiography system. All measurements were averaged on 10 consecutive cardiac cycles per experiment and were analyzed by one experienced investigator.

#### Electrocardiography

Electrocardiography (ECG) was performed under isoflurane (1.5%) anesthesia. Mice were placed on a thermocontrolled plate (37±1°C) and were given 10 minutes to acclimate before ECG recording. Signal-averaged ECG tracings were obtained by subcutaneous placement of 27-gauge steel needle electrodes in each limb, secured with tape. ECG was recorded for 60 minutes with the PowerLab Chart 7.1 system (ADInstruments, Sydney, Australia) and then was analyzed offline.

#### Angiogenic Response After Peripheral Chronic Ischemia

Peripheral chronic ischemia was induced in 6-month-old mice by means of surgical ligation and excision of the right common femoral artery. We have published a detailed description of the procedure.^21–23^ The angiogenic response was assessed on postoperative days 3, 7, 14, and 21 by laser Doppler (Perimed Instruments, Järfälla, Sweden).^22^ Furthermore, 3 weeks after surgery, we performed (1) ultrasound Doppler analysis of the posterior tibial artery with a VeVo770 imaging system equipped with a 20- to 60-MHz scanhead (VisualSonics, Inc, Toronto, Canada), (2) dyed-microbead assay on the gastrocnemius muscle, and (3) histological analysis of the anterior tibial muscle.

#### Myocardial Infarction

Reproducible infarcts of the anterior LV wall were imposed on 6-month-old mice by cryogeny with a 6-mm^2^ cryoprobe. Briefly, after isofluorane (2%) anesthesia, a thoracotomy was performed through the fourth left intercostal space, the pericardium was opened, and the heart was exposed. Cryoinfarction was produced by applying the cryoprobe to the anterior LV free wall, followed by freezing for 10 seconds. The exact position of the probe was set carefully by using the left atrium and pulmonary artery as anatomic landmarks. Rinsing with room-temperature saline was performed to allow nontraumatic detachment of the probe from the LV wall after freezing. Cardiac ultrasound analysis was performed 8 weeks after the lesion was created.

#### Urinary Protein Excretion

As a marker of renal damage,^16^ we assessed urinary protein excretion nephelometry (bicinchoninic acid method; Pierce, Rockford, IL) by placing the mice (n=12 in each group) in metabolic cages (Tecniplast, Buguggiate, Italy) for 24 hours.

### Ex Vivo Studies

#### Vascular Reactivity

Aortic rings (6 to 9 mm) from 6-month-old mice were suspended in isolated tissue baths (Radnoti Glass Technology, Monrovia, CA) filled with 25 mL Krebs-Henseleit solution (in mmol/L: NaCl 118.3, KCl 4.7, CaCl_2_ 2.5, MgSO_4_ 1.2, KH_2_PO_4_ 1.2, NaHCO_3_ 25, and glucose 5.6) continuously bubbled with a mixture of 5% CO_2_ and 95% O_2_ (pH 7.38 to 7.42) at 37°C, according to the protocol used in our laboratory.^23^ Vasorelaxation was assessed in vessels preconstricted with phenylephrine (1 μmol/L) in response to isoproterenol, acetylcholine, or nitroprusside, all from 10 nmol/L to 10 μmol/L, freshly prepared on the day of experiment.^23^ Endothelium-independent vasorelaxation also was tested after incubation (10 μmol/L, 15 minutes) with *N*^G^-nitro-l-arginine methyl ester (Sigma-Aldrich, Milan, Italy), a competitive inhibitor of endothelial nitric oxide synthase (eNOS). Concentrations are reported as the final molar value in the organ bath.

#### Histology

Samples (hearts, kidneys, muscles) were fixed in 10% buffered formalin and processed for paraffin embedding.^24^ Slides were stained with hematoxylin and eosin for architectural analysis or with Masson's trichrome to assess the presence and extent of interfiber interstitial fibrosis.^19,24–25^ Percent collagen was calculated from high-resolution, color-calibrated digital images of Masson's trichrome–stained sections with the use of dedicated software (NIH ImageJ64), as described.^19^ To measure myocyte cross-sectional area (μm^2^) we used fluorescence-tagged wheat germ agglutinin staining (5.0 μg/mL; with samples incubated in the dark for 10 minutes at 37°C). Images were recorded at 494-nm excitation and 518-nm emission and were evaluated with ImageJ64. Lectin immunohistochemical staining was performed on myocardial and skeletal muscle.^21,26^

#### Quantification of Atherosclerotic Lesions

Atherosclerotic lesions in 18-month-old mice were detected by staining with the neutral lipid-targeting lysochrome Oil Red O (Sigma-Aldrich, Milan, Italy). Each aorta was rinsed first in distilled water and then quickly in 60% isopropyl alcohol. Subsequently, vessels were stained for 25 minutes (in a solution of 15 g Oil Red O, 30 mL isopropyl alcohol, and 20 mL distilled water, freshly prepared and filtered) and then were washed. The Oil Red O–stained areas of the inner aortic surfaces were quantified using the free software Fiji. The extent of atherosclerosis was assessed on longitudinally opened aorta and expressed as the percentage of the lipid-accumulating lesion area to the total aortic area analyzed. Acquisition of images and analysis of lesions were performed in a blinded fashion.

### In Vitro Assays

#### Cell Culture

Murine aortic ECs were isolated from 3-month-old *CaMK4*^−/−^ and *CaMK4*^+/+^ animals as previously described.^27^ Bovine aortic ECs and human embrionic kidney (HEK293) cells were purchased from Lonza (Basel, Switzerland) and American Type Culture Collection (ATCC; Manassas, VA), respectively. Cells were cultured in Dulbecco's modified Eagle medium (Sigma-Aldrich, Milan, Italy) as described.^19^ All experiments were performed in triplicate to ensure reproducibility. We used ionomycin (1 μmol/L; Sigma-Aldrich) as activator and KN93 (5 μmol/L; Seikagaku Corporation, Tokyo, Japan) as inhibitor of CaMK.^5,28^

#### Immunoprecipitation and Immunoblotting

Immunoblot analysis was performed as previously described and validated.^21^ Blots were probed with mouse monoclonal antibodies against eNOS, phospho-eNOS (peNOS Ser^1177^), CaMKIV (BD Bioscience, Franklin Lakes, NJ), peNOS Ser^114^, peNOS Ser^615^ (Millipore, Billerica, MA), peNOS Thr^495^, pCaMKIV, CaMKII, pCaMKII, and actin (Santa Cruz Biotechnology, Santa Cruz, CA). Images then were digitalized and densitometry was assessed with dedicated software (Image Quant, GE Healthcare, Piscataway, NJ). Data are presented as arbitrary units after normalization for the total corresponding protein or actin as loading control, as indicated.

#### eNOS Activity Assay

eNOS activity was detected in *CaMK4*^−/−^ and *CaMK4*^+/+^ murine aortic ECs and in bovine aortic ECs by measuring the conversion of l-[^3^H]arginine to l-[^3^H]citrulline at 37°C for 30 minutes with the eNOS assay kit (Calbiochem-Nova Biochem, San Diego, CA), according to the manufacturer's instructions. Unlabeled l-arginine was added to l-[^3^H]arginine (specific activity, 60 Ci/mmol/L) at a ratio of 3:1. Mouse cerebellum extracts, containing elevated amounts of neuronal NOS, were used as positive controls, whereas samples incubated in the presence of *N*^G^-nitro-l-arginine methyl ester (1 mmol/L) were used to determine nonspecific activity.

#### CaMKIV Activity Assay

The CaMKIV activity assay consisted of 2 reaction steps. Briefly, in the first step, active recombinant full-length CaMKIV (Millipore, Billerica, MA) was incubated at 30°C for 30 minutes with 0.5 mmol/L CaCl_2_ and 1 μmol/L CaM in 25 μL of a reaction mixture (25 mmol/L 4-[2-hydroxyethyl]-1-piperazineethanesulfonic acid [HEPES], pH 7.5, 0.5 mmol/L MgCl_2_, 1 mmol/L dithiothreitol, 0.5 mg/L BSA, 1 mmol/L sodium orthovanadate, 0.1 mmol/L cold adenosine triphosphate (ATP), and H_2_O 0.01% Tween 20). In the second step, a 20-μL aliquot from the first reaction mixture containing active CaMKIV was incubated in 50 μL of a solution containing 0.1 IU of eNOS (Calbiochem-Nova Biochem, San Diego, CA) as substrate and 1 mmol/L EGTA, 0.5 μL [^32^P]-γATP (3000 Ci/mmol/L) for 30 minutes at 30°C. The reactions were stopped by the addition of sodium dodecyl sulfate–polyacrylamide gel electrophoresis (SDS-PAGE) sample loading buffer, and the whole reaction mixes were separated on 4% to 12% SDS-PAGE (Life Technologies, Grand Island, NY). Then, the gel was dried and peNOS was visualized by phosphorImager (GE Healthcare, Piscataway, NJ).

Alternatively, a partially modified protocol was performed without the use of radioactive ATP. In this case, after PAGE, proteins were blotted on nitrocellulose, and eNOS phosphorylation by CaMKIV was also assessed by Western blot by using the previously mentioned antibodies.

#### Overlay Blot Assay

Twenty nanograms of CaMKIV and 0.1 IU of eNOS-purified proteins were subjected to SDS-PAGE and transferred on nitrocellulose. The membranes were incubated 2 hours at room temperature in 5% blocking solution. At this time, the filters were incubated with CaMKIV or eNOS-purified protein in phosphorylation solution (final concentrations: 20 μmol/L ATP, 1 mmol/L CaCl_2_, 20 mmol/L MgCl_2_, and 4 mmol/L Tris, pH 7.5). After 1 hour of incubation at room temperature, the blots were cooled rapidly on ice; washed twice with NaCl, Tris, and 0.1% Tween 20; and then fixed with 0.5% formaldehyde for 10 minutes. The filters were washed 3 times with 2% glycine and once with NaCl, Tris, and 0.1% Tween 20. CaMKIV or eNOS-bound proteins were detected by chemiluminescence.^29^

### Human Association Study

Study participants were consecutive hypertensive patients referred to the Hypertension Diagnosis and Care Outpatient Clinic of “Federico II” University of Naples. Age-matched unaffected controls were recruited from a database of normotensive blood donors. The matching design was accounted for in the statistical analyses. All subjects were white and born within the Campania region in Southern Italy. We had access to a digital archive for each participant.^30^ Enrollment criteria for hypertensive status were an age of 18 to 80 years and a confirmed diagnosis of essential hypertension. We considered the average systolic and diastolic BP values, according to European guidelines.^16^

Analysis of the rs10491334 SNP of *CaMK4* was performed on peripheral blood DNA by restriction fragment length polymorphism.^30^ Genetic analyses were performed by laboratory personnel blinded to sample identity. Patients' lymphocytes were extracted by means of phicoll purification with HISTOPAQUE-1077 (Sigma-Aldrich) from a 20-mL blood sample, which had been anticoagulated with ethylenediaminetetraacetic acid.^31^

### Data Presentation and Statistical Analysis

Data are presented as mean ± standard error (SE) unless otherwise mentioned. To determine the statistical significance of the results, we used 1-way ANOVA or Kruskal-Wallis test, as appropriate (nonparametric analysis was used when a large number of tests increased the risk of a type α error). Survival curves were compared with the log-rank test.

To assess significant differences between genotype classes in the human studies, we used the Student *t* test for continuous variables and the Fisher exact test for categorical variables. The association between the SNP and hypertension was adjusted for age, sex, heart rate, and body mass index. Statistical significance was set at *P*<0.05. All the analyses were performed with GraphPad Prism version 5.01 (GraphPad Software, San Diego, CA), Systat 13 (Systat software, Inc, Chicago, IL), and Statistical Package for Social Sciences software version 20.0.0 (IBM SPSS Inc, Armonk, NY).

## Results

### BP, Organ Damage, and Survival

**Figure 1.**
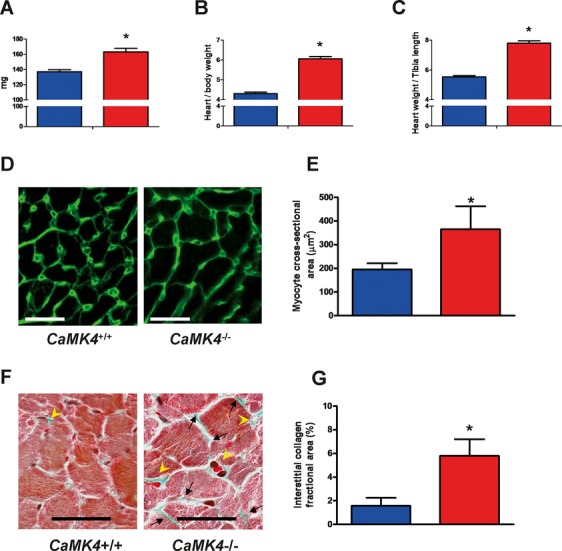
LVH in *CaMK4*^−/−^ mice. *CaMK4*^−/−^ 6-month-old mice present cardiac hypertrophy, as determined by an increase in heart weight (A), normalized by body weight (B) or tibial length (C). Fluorescence-tagged wheat germ agglutinin, which binds to saccharides of cellular membranes, showed increased cardiac myocyte cross-sectional area in *CaMK4*^−/−^ mice (D and E; magnification ×200, scale bar = 15 μm). Masson's trichrome staining (F; magnification ×300, scale bar = 15 μm; interstitial and perivascular collagen deposition indicated by black arrows and yellow arrowheads, respectively) also revealed an increase in fibrosis, quantified as described in Methods (G). Blue bars are *CaMK4*^+/+^ mice; red bars, *CaMK4*^−/−^ mice. *P<0.05 vs *CaMK4*^+/+^.

**Figure 2.**
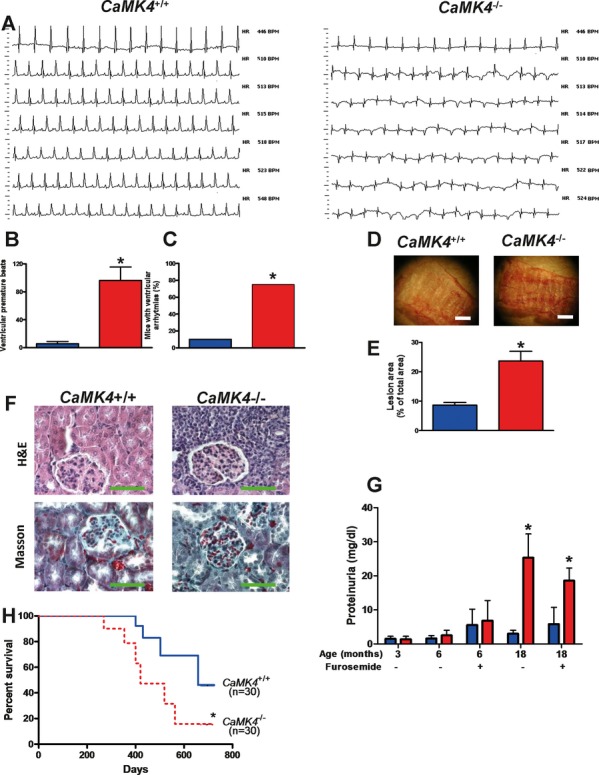
Organ damage in 18-month-old mice. Old *CaMK4*^−/−^ mice displayed a dilatation of the LV (see also Table 1) and exhibited several spontaneous ventricular arrhythmias (A; representative ECG records at different heart rates). These data are quantified in B and C (n=10 per group; *P<0.05). Of note, heart rate among mutated animals did not significantly differ (see Table 1). D and E, Lipid deposition in vessels of *CaMK4*^+/+^ and *CaMK4*^−/−^ mice (n=12 per group). Representative pictures of oil red O–stained ascending aortas (D; white bar = 600 μm); areas of atherosclerotic lesions were quantified using the free software Fiji and are represented as percentage of lesion area to total aortic area (E; *P<0.05 vs *CaMK4*^+/+^). Kidneys (F) from the *CaMK4*^−/−^ group exhibited increased glomerulosclerosis, inflammatory cell infiltration, and tubulointerstitial fibrosis compared with *CaMK4*^+/+^ mice (n=8 per group; representative pictures of hematoxylin and eosin or Masson's trichrome staining; magnification ×60; green bar = 100 μm). Moreover, 18-month-old *CaMK4*^−/−^ mice presented another typical feature of the hypertensive phenotype (G), showing greater proteinuria than *CaMK4*^+/+^ mice (n=12 per group; *P<0.05 vs *CaMK4*^+/+^). In all histograms, blue bars are *CaMK4*^+/+^ mice; red bars, *CaMK4*^−/−^ mice. Notably, compared with *CaMK4*^+/+^ mice, *CaMK4*-null mice displayed significantly (*P<0.05) impaired survival, as well (Kaplan-Meier curves; H). Blue line indicates *CaMK4*^+/+^ mice; red dotted line, *CaMK4*^−/−^ mice.

*CaMK4*^−/−^ mice developed higher systolic and diastolic BP levels than did *CaMK4*^+/+^ littermates, as shown in Table 1. We decided to assess BP-dependent phenotypes, such as target-organ damage, in homozygous mice only. At 6 months, cardiac ultrasound analysis showed that *CaMK4*^−/−^ mice displayed concentric^25,31–32^ LV hypertrophy (LVH) due to increased septum and posterior wall thickness, with no significant changes in internal diameter, as depicted in Table 1. At gross analysis, *CAMK4*^−/−^ hearts were larger both as absolute values (163.1±4.5 versus 136.8±3.1 mg, Figure 1A) and after correction by either body weight (Figure 1B) or tibial length (Figure 1C). Histological analysis showed increased cell size of *CAMK4*^−/−^ cardiomyocytes (Figure 1D and 1E) and augmented interstitial fibrosis (Figure 1F and 1G), 2 common elements of hypertension-induced LVH.^19^ In older mice (18 months old), cardiac damage evolved toward dilatation and dysfunction (Table 1) complicated by arrhythmias, as shown by ECG (Figure 2A through 2C), according to the natural history of untreated hypertension.^33^ Indeed, LVH is associated with alterations in the dispersion of repolarization^34^ and prolongation of ventricular action potentials.^35^ These effects result in electrical instability and increase the propensity to develop arrhythmias. When an antihypertensive treatment with furosemide was initiated at 3 months of age, the development of both hypertension and LVH was prevented (Table 2).

**Table 1.** Systemic and LV Hemodynamics in *CAMK4*^+/+^ and *CAMK4*^−/−^ Mice

	*CaMK4*^+/+^ (n=12)	*CaMK4*^−/−^ (n=13)	*CaMK4*^+/+^ (n=14)	*CaMK4*^−/−^ (n=16)	*CaMK4*^+/+^ (n=12)	*CaMK4*^−/−^ (n=13)
	3-Month-Old Mice		6-Month-Old Mice		18-Month-Old Mice	
HR, bpm	492±28	486±36	486±18	474±16	460±32	468±28
SBP, mm Hg	111±1.3	113±1.8	110±0.6	123±0.7*	111±1.1	124±1.3*
DBP, mm Hg	81±0.9	82±1.1	81±0.3	90±0.3*	86±0.8	93±0.9*
LVEDD, mm	3.71±0.42	3.73±0.35	3.85±0.32	3.74±0.29	3.89±0.37	4.36±0.3*
LVESD, mm	2.08±0.26	2.15±0.28	2.19±0.27	2.37±0.28	2.22±0.3	3.02±0.46*
IVS, mm	0.72±0.04	0.73±0.05	0.72±0.03	0.88±0.02*	0.82±0.04	0.89±0.03*
LVPW, mm	0.71±0.03	0.72±0.04	0.69±0.02	0.89±0.03*	0.78±0.03	0.92±0.04*
LVFS, %	43.4±4.8	42.6±5.5	42.4±4.5	36.2±6.1*	37.2±5.8	30.2±7.2*
LVEF, %	66.8±8.2	66.3±9.1	64.8±8.6	59.6±7.8	56.4±9.4	50.1±9.8*
+d*P*/d*t*, mm Hg/s	6334±602	6211±522	6237±531	5681±498*	6004±582	5014±578*
−d*P*/d*t*, mm Hg/s	6420±542	6322±508	6302±504	5702±486*	6080±682	5112±606*

Data are mean±SE. HR indicates heart rate; SBP, systolic BP; DBP, diastolic BP; LVEDD, LV end-diastolic diameter; LVESD, LV end-systolic diameter; IVS, interventricular septum; LVPW, LV posterior wall; LVFS, LV fractional shortening; and LVEF, LV ejection fraction.

* *P<*0.05 comparing *CaMK4*^−/−^ to *CaMK4*^+/+^at each time point; otherwise, *P* not significant.

**Table 2.** Effect of Long-Term Diuretic Treatment on Systemic and LV Hemodynamics in *CAMK4*^+/+^ and *CAMK4*^−/−^ Mice

	*CaMK4*^+/+^ (n=11)	*CaMK4*^−/−^ (n=16)	*CaMK4*^+/+^ (n=10)	*CaMK4*^−/−^ (n=14)
	6-Month-Old Mice Treated (3 Months) With Furosemide		18-Month-Old Mice Treated (15 Months) With Furosemide	
HR, bpm	498±46	492±38	483±52	488±41
SBP, mm Hg	107±6.8	114±1.8	103±6.6	104±1.3
DBP, mm Hg	74±8.5	83±1.1	75±5.9	78±1.2
LVEDD, mm	3.82±0.9	3.83±0.4	3.9±0.8	3.95±0.7
LVESD, mm	2.21±0.8	2.23±0.3	2.26±0.9	2.28±0.5
IVS, mm	0.71±0.08	0.76±0.04	0.78±0.09	0.8±0.05
LVPW, mm	0.69±0.07	0.77±0.05	0.76±0.08	0.79±0.06
LVFS, %	41.6±8.8	39.2±7.3	36.9±8.6	36.5±7.1
LVEF, %	62.8±9.7	62.5±8.4	55.4±8.1	55.1±8.9
+d*P*/d*t*, mm Hg/s	6126±684	6088±642	5522±528	5876±627
−d*P*/d*t*, mm Hg/s	6188±747	6104±594	5596±639	5892±582

Data are mean±SE. HR indicates heart rate; SBP, systolic BP; DBP, diastolic BP; LVEDD, LV end-diastolic diameter; LVESD, LV end-systolic diameter; IVS, interventricular septum; LVPW, LV posterior wall; LVFS, LV fractional shortening; and LVEF, LV ejection fraction.

Along with increased heart size, *CAMK4*^−/−^ mice over time developed hypertensive vascular damage, as assessed by Oil Red O staining (Figure 2D), which revealed larger atherosclerotic lesions in aortas from 18-month-old *CaMK4*^−/−^ mice versus *CaMK4*^+/+^ mice (Figure 2E). We also found renal damage, another typical feature of the hypertensive phenotype,^16^ inasmuch as *CaMK4*^−/−^ mice displayed increased glomerulosclerosis, inflammatory cell infiltration, and tubulointerstitial fibrosis compared with *CaMK4*^+/+^ mice (Figure 2F). The functional correlate to this histological alteration is increased proteinuria in 18-month-old *CaMK4*^−/−^ mice (Figure 2G). Furosemide-treated *CaMK4*^−/−^ mice still presented proteinuria (Figure 2G), although they had normal BP levels.

Eventually, *CaMK4*^−/−^ mice showed significantly reduced lifespan compared with *CAMK4*^+/+^ littermates (Figure 2H).

### Assessment of Endothelium-Dependent Phenotypes

**Figure 3.**
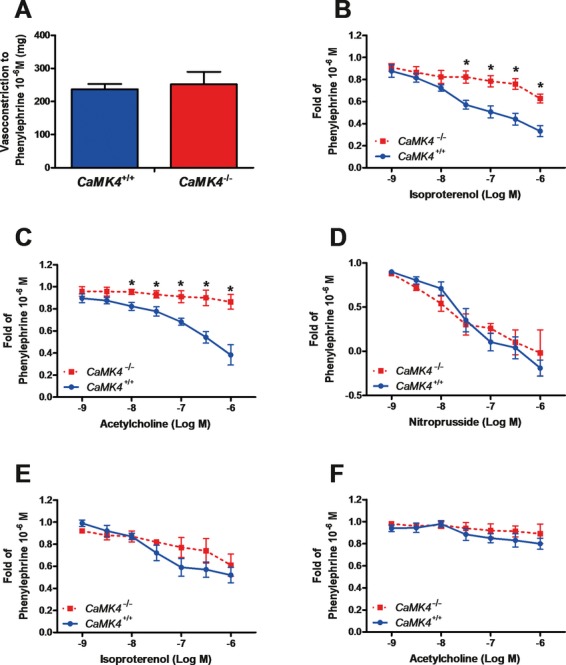
Vascular responses on isolated aortic rings from 6-month-old mice. Vasoconstriction to α_1_-adrenergic agonist phenylephrine was similar in *CaMK4*^+/+^ and *CaMK4*^−/−^ mice (A). Endothelium-dependent vasorelaxation induced by the β-adrenergic agonist isoproterenol (B) or by the muscarinic agonist acetylcholine (C) was blunted in *CaMK4*^−/−^ vessels, whereas endothelium-independent vasodilation to nitroprusside was not different between *CaMK4*^+/+^ and *CaMK4*^−/−^ (D). To better explore the role of nitric oxide in endothelial responses, we also evaluated vascular responses in the presence (10 μmol/L) of the specific eNOS inhibitor *N*^G^-nitro-l-arginine methyl ester (E and F). *P<0.05 vs *CaMK4*^+/+^.

Endothelium-dependent vasodilation in ex vivo experiments was assessed on isolated aortic rings. After vasoconstriction obtained through 1 μmol/L phenylephrine (Figure 3A), aortic rings from *CaMK4*^−/−^ mice showed impaired endothelial-dependent vasodilation both to the β-adrenergic agonist isoproterenol and to the muscarinic agonist acetylcholine, which is consistent with our hypothesis of endothelial dysfunction (Figure 3B and 3C), a known hallmark of the hypertensive state.^13^ Moreover, there was no difference in the vascular smooth muscle cell–mediated response to the nitric oxide donor nitroprusside (Figure 3D), and experiments performed after *N*^G^-nitro-l-arginine methyl ester incubation confirmed intact endothelium-independent vasodilation in both *CaMK4*^−/−^ and *CaMK4*^+/+^ vessels (Figure 3E and 3F).

**Figure 4.**
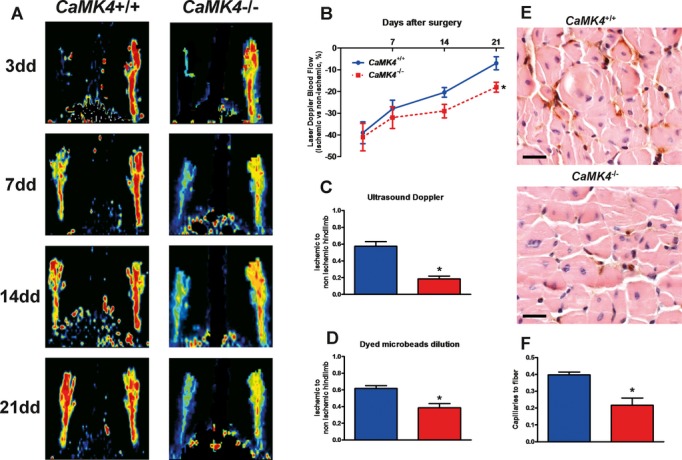
Decreased neoangiogenic responses in *CaMK4*^−/−^ mice during chronic ischemia in vivo (n=6 per group). Determination of laser Doppler blood flow (A and B) on postoperative days 3, 7, 14, and 21 showed a deficit in ischemic hindlimb perfusion, compared with the contralateral hindlimb, that was significantly increased in *CaMK4*^−/−^ vs *CaMK4*^+/+^ mice (*P<0.05, repeated measurements, ANOVA; laser Doppler blood flow data are expressed as percent of ischemic to nonischemic limb). Ultrasound Doppler flowmetry of posterior tibial artery (C), performed 3 weeks after femoral artery removal, confirmed the attenuated blood flow restoration in *CaMK4*^−/−^ mice (*P<0.05 vs *CaMK4*^+/+^). This result was mirrored by the dyed-bead perfusion analysis (D; *P<0.05 vs *CaMK4*^+/+^). Lectin staining of capillaries in the ischemic hindlimb (E; magnification ×20, black bar = 100 μm) showed that chronic ischemia produced a greater rarefaction of the capillary density, evaluated as number of capillaries corrected for the number of muscle fibers (F), in *CaMK4*^−/−^ compared with *CaMK4*^+/+^ mice (*P<0.05). In all histograms, blue bars are *CaMK4*^+/+^ mice; red bars, *CaMK4*^−/−^ mice.

Additionally, we assessed in vivo the angiogenic response to ischemia after femoral artery removal, a phenotype that is largely under the control of the endothelium.^21,23,36^ *CaMK4*^−/−^ mice displayed impaired angiogenesis after 21 days of peripheral ischemia, as assessed by laser Doppler (Figure 4A and 4B), Doppler ultrasound (Figure 4C), dyed microbeads (Figure 4D), and capillary density (Figure 4E and 4F).

### Development of Heart Failure After Myocardial Infarction

**Figure 5.**
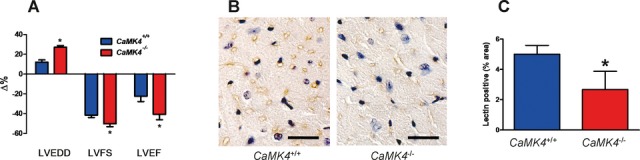
Cardiac evaluation after cryoinjury. Changes in echocardiographic parameters (A) 8 weeks after myocardial infarction. (LVEDD indicates LV end-diastolic diameter; LVFS, LV fractional shortening; and LVEF, LV ejection fraction. n=12 per group. *P<0.05 vs *CaMK4*^+/+^). Immunohistochemical analysis (B; lectin staining, magnification ×60, black bar = 30 μm) of myocardium in mice 8 weeks after infarction. The peri-infarct area demonstrated lower capillary density, as confirmed by quantitative analysis (C); blue bars are *CaMK4*^+/+^ mice; red bars, *CaMK4*^−/−^ mice. *P<0.05 vs *CaMK4*^+/+^.

Increased BP and reduced angiogenesis are expected to precipitate the evolution of the heart failure phenotype after myocardial damage.^17,37^ Indeed, 8 weeks after myocardial cryoinfarction, *CaMK4*^−/−^ mice presented larger LV dilatation and a greater decrease in cardiac function than did *CAMK4*^+/+^ mice, as shown in both Table 3 and Figure 5A. This feature also was accompanied by reduced capillary density in the peri-infarct area (Figure 5B and 5C).

**Table 3.** Systemic and LV Hemodynamics in *CAMK4*^+/+^ and *CAMK4*^−/−^ Mice 8 Weeks After Myocardial Infarction

	*CaMK4*^+/+^ (n=12)	*CaMK4*^−/−^ (n=14)
HR, bpm	498±20	482±18
SBP, mm Hg	100±0.5	108±0.6*
DBP, mm Hg	74±0.3	82±0.4*
LVEDD, mm	4.16±0.12	4.6±0.11*
LVESD, mm	3.15±0.08	3.71±0.13*
IVS, mm	0.66±0.04	0.85±0.05*
LVPW, mm	0.68±0.03	0.86±0.03*
LVFS, %	24.74±2.9	17.8±2.3*
LVEF, %	49.02±4.8	35.8±3.4*
+d*P*/d*t*, mm Hg/s	3784±402	3004±382*
−d*P*/d*t*, mm Hg/s	3918±386	3128±394*

Data are mean±SE. Mice were 8 months old (myocardial infarction was induced in 6-month-old animals). HR indicates heart rate; SBP, systolic BP; DBP, diastolic BP; LVEDD, LV end-diastolic diameter; LVESD, LV end-systolic diameter; IVS, interventricular septum; LVPW, LV posterior wall; LVFS, LV fractional shortening; and LVEF, LV ejection fraction.

* *P*<0.05; otherwise, *P* not significant.

### Interaction Between CaMKIV and eNOS

**Figure 6.**
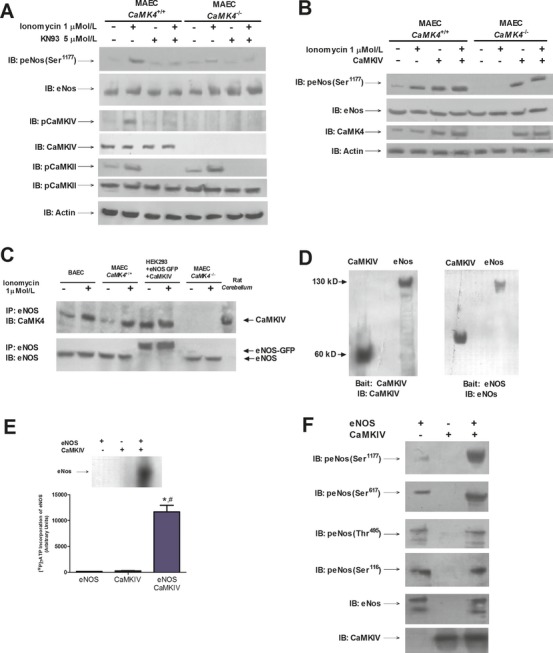
Interaction between CaMKIV and eNOS. eNOS phosphorylation (Ser^1177^) is enhanced by ionomycin, echoing the phosphorylation of CaMKIV, and is prevented by the CaMK inhibitor KN93 (A). Notably, eNOS activation was less evident in *CaMK4*^−/−^ MAEC, where *CaMK4* was not expressed (A). Transgenic restoration of CaMKIV expression in *CaMK4*^−/−^ ECs corrected calcium-induced eNOS activation (B). The interaction between CaMKIV and eNOS was demonstrated by performing immunoprecipitation (IP) experiments in different cellular settings, both in basal conditions and after stimulation with ionomycin (C). Such interaction is shown in BAEC and *CaMK4*^+/+^ MAEC but not in *CaMK4*^−/−^ MAEC. In a nonendothelial cell type, HEK293, we confirmed the interaction after reconstituting the system by using a plasmid encoding CaMKIV and a plasmid encoding eNOS linked to GFP (C; rat cerebellum was used as CaMKIV-positive control). The input protein levels are shown in Figure 8. Overlay assay with purified CaMKIV (left blot) or eNOS (right blot) as bait (D). CaMKIV induced eNOS [^32^P]-γATP incorporation (E). Purified CaMKIV induced eNOS phosphorylation on Ser^1177^ and Ser^615^ but not on Ser^114^ and Thr^495^ (F). *P<0.05 vs eNOS, *P<0.05 vs CaMKIV; representative images from triplicate experiments are shown. Densitometric analyses are reported in Figure 7. MAEC indicates murine aortic ECs; BAEC, bovine aortic ECs.

**Figure 7.**
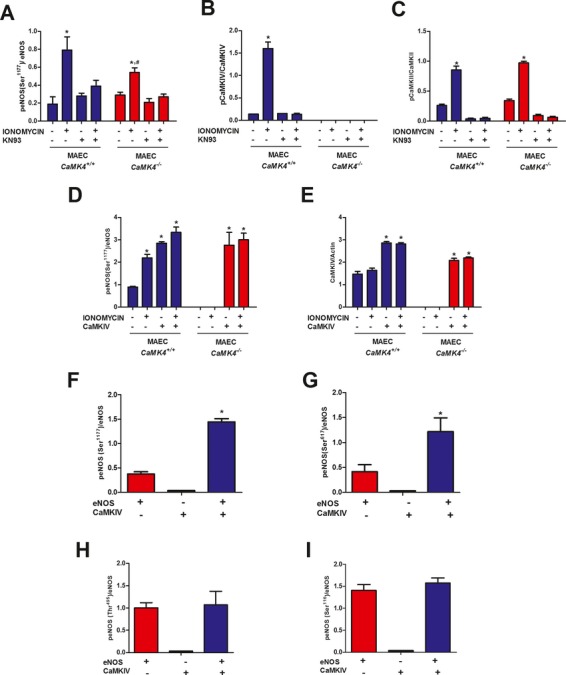
Quantification of blot results presented in Figure 6. Relative protein quantification levels for Figure 6A (A–C) and Figure 6B (D–E). Blue bars are *CaMK4*^+/+^; red bars, *CaMK4*^−/−^. *P<0.05 vs untreated cells; #*P*<0.05 vs MAEC *CaMK4*^+/+^. MAEC indicates murine aortic ECs. Densitometric analyses for Figure 6F (F–H) showing CaMKIV-mediated eNOS phosphorylation on Ser^1177^ and Ser^615^ but not on Ser^114^ and Thr^495^. Red bars indicate eNOS; black bars, CaMKIV; and blue bars, eNOS and CaMKIV. *P<0.05 vs eNOS.

**Figure 8.**
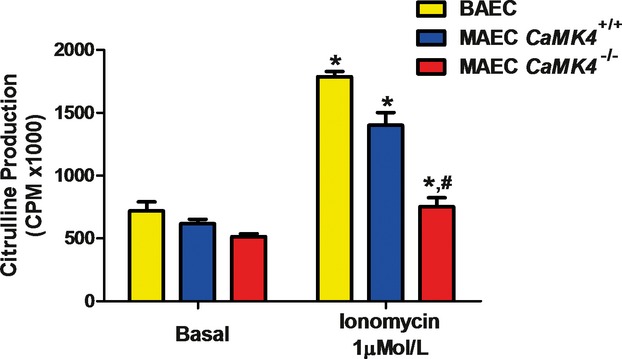
eNOS activity in ECs. eNOS activity, assessed by arginine–citrulline conversion, after stimulation by ionomycin (1 μmol/L) was blunted in *CaMK4*^−/−^ MAEC. *P<0.05 vs basal; #*P*<0.05 vs *CaMK4*^+/+^. MAEC indicates murine aortic ECs; CPM, counts per minute.

**Figure 9.**
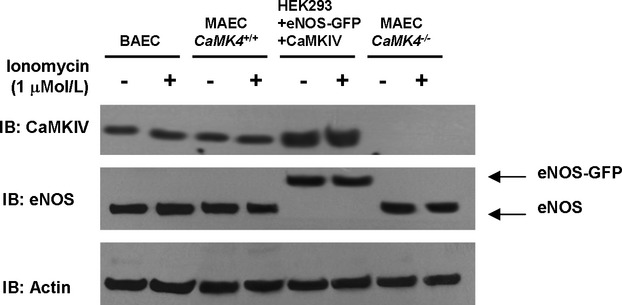
Input Western blots of immunoprecipitation assay represented in Figure 6C. To confirm that equal amounts of proteins were present in the cell lysates used for immunoprecipitation as depicted in Figure 6C, we performed Western blotting on 30 μg of proteins of corresponding cell lysates with the same antibodies used in the experiment represented in Figure 6C, raised respectively against CaMKIV and eNOS. Furthermore, actin was detected to confirm equal amount of proteins.

Because endothelium dysfunction can be mimicked by altered production or removal of nitric oxide, we assessed the effect of *CaMK4* knockout on eNOS activation. Indeed, calcium-induced eNOS phosphorylation on Ser^1177^ is impaired in *CaMK4*^−/−^ ECs. (Figures 6A and 7A through 7C). This impairment associates with a reduction in CaMKIV expression and activity in *CaMK4*^−/−^ EC, whereas activation of CaMKII remains unaffected (Figure 6A). This finding also is endorsed by a reduction in eNOS activity (Figure 8). Interestingly, transgenic restoration of CaMKIV expression in *CaMK4*^−/−^ ECs also corrects calcium-induced eNOS activation (Figures 6B, 7D, and 7E). CaMKIV and eNOS can coimmunoprecipitate in either naïve or overexpressing cells (Figures 6C and 9). The physical interaction between the 2 proteins can be replicated in an overlay assay with the use of purified CaMKIV and eNOS, which indicates that the interaction is not mediated by a third component (Figure 6D). The result of this physical interaction is the incorporation in eNOS of ^32^P (Figure 6E). Also, in vitro, purified CaMKIV can phosphorylate eNOS directly on Ser^1177^ and Ser^615^, 2 phosphorylation sites that are regulatory for enzyme activity, but not on other phosphorylation sites of eNOS, namely Ser^114^ and Thr^495^ (Figures 6F and 7F through 7I).

### Role of rs10491334 T/C Polymorphism of Human *CaMK4* Gene in Hypertensive Patients

**Figure 10.**
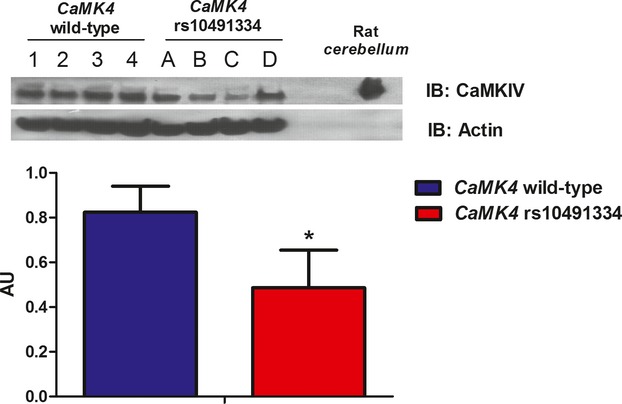
Expression levels of CaMKIV in circulating lymphocytes of hypertensive patients. Western blot analysis of CaMKIV on peripheral blood lymphocytes showed that CaMKIV levels were higher in subjects with the *CaMK4* wild-type genotype (1, 2, 3, and 4 represent samples from 4 different individuals) than in subjects homozygous for the polymorphic *CaMK4* rs10491334 variant (A, B, C, and D represent samples from 4 different individuals). Rat cerebellum was used as CaMKIV-positive control. Data from immunoblots (IB; representative images from 5 experiments are shown) were quantified by densitometric analysis. CaMKIV levels were normalized to actin densitometry. *P<0.05 vs *CaMK4*^+/+^. AU indicates arbitrary units.

The results gained in the *CaMK4*^−/−^ mouse show a role for this kinase in the setup of hypertension. To confirm the possible relevance of this finding in humans, we studied the frequencies of the rs10491334 T/C SNP.^6^ This polymorphism associates with a reduction in the cellular expression of the kinase.^38^ We studied 2 populations of normotensive (n=457) and hypertensive subjects (n=730). Clinical characteristics of these individuals are reported in Table 4. We found a higher occurrence of the polymorphism among the hypertensive patients that fell short of statistical significance (normotensive patients: 31.32%; hypertensive patients: 41.64%; *P=*0.0594, Pearson χ^2^ analysis). Thus, we dichotomized our hypertensive population according to European Guidelines into categories of severe (Grade 2/3) and not-severe (Grade 1) diastolic hypertension (cutoff: diastolic BP=100 mm Hg)^16^ and found a significantly larger frequency of the T variant of the rs10491334 SNP in patients with severe hypertension than in patients with diastolic BP<100 mm Hg (54.42% versus 38.41%; *P*<0.05, Pearson χ^2^ analysis), as shown in Table 5. Intriguingly, hypertensive patients homozygous for the polymorphic T allele showed reduced expression levels of CaMKIV in circulating peripheral blood lymphocytes (Figure 10).

**Table 4.** Characteristics of the Normotensive and Hypertensive Populations

	Normotensive Subjects (n=457)	Hypertensive Subjects (n=730)
Age, y	52.91±3.4	55.84±2.9
Sex (male/female), n	288/169	452/278
HR, bpm	76.8±9.3	72.8±8.2
SBP, mm Hg	131.67±0.84	147.7±0.72*
DBP, mm Hg	77.06±0.51	96.69±0.48*
Body mass index, kg/m^2^	26.03±0.27	27.19±0.21
Smoking (current or former), %	53.6	54.1
Glycemia, mmol/L	4.8±0.72	5.2±0.88
Diabetes, %	4.7	5.9
Total cholesterol, mmol/L	4.35±0.12	4.98±0.18
High-density lipoprotein cholesterol, mmol/L	1.24±0.11	1.21±0.14
Low-density lipoprotein cholesterol, mmol/L	2.96±0.13	3.25±0.19
Triglycerides, mmol/L	1.46±0.09	1.53±0.11
Dyslipidemia, %	37.8	41.2
LV mass index, g/m^2^	92.6±4.5	115.34±4.8*
*CaMK4* rs10491334 polymorphism (T-allele frequency), %	31.32	41.64

Data are mean±SE; n, or %, as indicated. HR indicates heart rate; SBP, systolic BP; and DBP, diastolic BP.

* *P*<0.05; otherwise, *P* not significant.

**Table 5.** Characteristics of the Hypertensive Patients, Subdivided Into 2 Populations According to a DBP Cutoff of 100 mm Hg

	Patients With DBP <100 mm Hg (n=583)	Patients With DBP ≥100 mm Hg (n=147)
Age, y	54.9±3.6	56.2±3.8
Sex, male/female, n	371/212	81/66
HR, bpm	71.6±9.6	72.2±11.3
SBP, mm Hg	142.86±1.58	154.46±1.42*
DBP, mm Hg	91.89±0.74	108.69±0.86*
Body mass index, kg/m^2^	26.85±0.63	27.71±1.74
Smoking (current or former), %	55.4	53.3
Glycemia, mmol/L	5.4±0.77	5.1±0.98
Diabetes (%)	5.7	6.1
Total cholesterol, mmol/L	4.77±0.22	5.01±0.28
High-density lipoprotein cholesterol, mmol/L	1.22±0.15	1.17±0.19
Low-density lipoprotein cholesterol, mmol/L	3.32±0.17	3.05±0.23
Triglycerides, mmol/L	1.52±0.13	1.55±0.16
Dyslipidemia, %	43.1	39.8
LV mass index, g/m^2^	112.6±4.5	116.86±7.3
*CaMK4* rs10491334 polymorphism (T-allele frequency), %	38.41	54.42*

Data are mean±SE; n, or %, as indicated. HR indicates heart rate; SBP, systolic BP; and DBP, diastolic BP.

* *P<*0.05; otherwise, *P* not significant.

## Discussion

In the present study, we provide compelling evidence for a fundamental and previously unrecognized role of CaMKIV in the regulation of vascular function. Indeed, the phenotype of the *CaMK4*^−/−^ mouse indicates that this kinase is extremely important for endothelial function. A paramount finding of this study is that the loss of *CaMK4* results in the development of hypertension, accompanied by its typical hallmarks: endothelial dysfunction, target-organ damage, and reduced survival rate.^16,32^ Interestingly, furosemide-treated mice did not display LVH, which suggests that LVH is indeed the result of increased BP rather than being genetically determined by *CaMK4* gene removal.^13^ We used a loop diuretic to obtain an effective BP decrease with minimum effect on vascular function.^16^ Moreover, other diuretics, such as thiazides, have metabolic implications not present in furosemide treatment^16^ that could confuse the cardiovascular phenotype of our *CaMK4*^−/−^ mice further. In our model, endothelial dysfunction could be either primitive to hypertension or, alternatively, secondary to the hypertensive state of *CaMK4*^−/−^ mice. We rule out this second hypothesis on the basis of 2 pieces of evidence: First, diuretic treatment resulted in normalization of hemodynamic-dependent LVH but did not correct proteinuria, which is a characteristic of endothelial dysfunction.^39^ Second, in a hemodynamic-independent setup^36^ (ie, in isolated ECs), *CaMK4* removal causes endothelial dysfunction as assessed by reduction of eNOS activity, which is corrected only after gene replacement. Endothelial dysfunction is known to induce hypertension, as demonstrated in *eNOS*^−/−^ mice, which show absent endothelial-dependent vasorelaxation and increased BP.^13^

Our study is the first to demonstrate the interaction of CaMKIV and eNOS. Previous reports had suggested the importance of CaMKs in endothelium-dependent relaxation.^40–42^ In addition, 2 independent groups had provided evidence that a nonselective CaMK inhibitor could significantly decrease bradykinin-induced eNOS activity^12^ and prevent eNOS phosphorylation^28^ in rat and porcine aortic ECs, respectively. Our study characterizes the close relationship between CaMKIV and eNOS in the endothelium. Indeed, CaMKIV can phosphorylate eNOS directly in Ser^1177^ and Ser^615^, 2 sites that are known to induce eNOS activation. Although we have not investigated all phosphorylation sites of eNOS, these 2 appear to be relevant for the described mechanism.

It is remarkable that CaMKII does not seem to supersede CaMKIV loss. Although CaMKIV and CaMKII often are considered mutually exchangeable, these 2 kinases present differences in tissue distribution and regulation^43–44^ and cannot be considered isoforms.^5^ For the present study, we used mice generated in the Means' laboratory in the 1990s.^9^ The cardiovascular phenotype of this mouse was never before investigated as extensively as we have in the present report. A hint of the higher BP of *CaMK4*^−/−^ mice can be found in the article of Colomer and colleagues,^4^ showing that after constriction of the thoracic aorta, BP gradients were higher in *CaMK4*-null than in wild-type mice. Other reports have failed to describe cardiovascular parameters of these mice. Interestingly, though, *CaMK4*^−/−^ mice present cognitive disorders that are typical of patients in the advanced phases of untreated hypertension,^45^ such as erasure of long-term memory.^10^ This phenotype has been ascribed to the loss of *CaMK4* signaling in neurons of *CaMK4*^−/−^ mice but also can be worsened by chronic exposure to increased BP levels.

To find a correlation between our observations in this genetically modified mouse and the human condition, we took advantage of the DNA Bank associated with the Campania Salute database of >5000 hypertensive patients.^30^ Previously, the Framingham study had identified an association marker for high diastolic BP in the rs10491334 SNP of the human *CaMK4* gene.^6^ Our study confirms this finding: We performed an association analysis with a candidate gene approach and found a significant correlation between the rs10491334 SNP and diastolic BP levels among hypertensive patients. Furthermore, in the present work, we show that this polymorphism associates with a reduction in the cellular expression levels of CaMKIV, similar to that observed in other populations.^38^ These data are highly suggestive of the intrinsic regulatory nature of CaMKIV in hypertension.

Our study follows the groove of the identification of the physiological implications of CaMKs in the cardiovascular system.^1–3^ Some authors have investigated the effects of CaMKIV in the heart by overexpressing it in cardiomyocytes, leading to cardiac hypertrophy.^46^ This notion was challenged by a more recent study showing that mice null for *CaMK4* still developed LVH.^4^ Our data reconcile these opposing views by suggesting that dysfunctional CaMKIV, albeit not expressed in the heart, might partake in cardiac organ damage in the context of the hypertensive state.

## Conclusion

Our findings establish that CaMKIV plays a relevant role in the regulation of the vascular tone by a mechanism that involves eNOS activation through phosphorylative events. Impairment of CaMK-mediated activation of eNOS, as in *CaMK4* gene deletion, induces hypertension, as demonstrated by the fact that *CAMK4*^−/−^ mice display a hypertensive phenotype that leads to typical organ damage. Extending our observations to the clinical scenario, we show that in hypertensive patients a *CaMK4* polymorphism that causes reduced expression of the protein identifies a subset of patients with higher BP levels. Altogether, our results point to CaMKIV as a novel potential biological target for therapeutic interventions in hypertension.
